# Potential and pitfalls of XRF-CS analysis of ion-exchange resins in environmental studies

**DOI:** 10.1038/s41598-021-00446-9

**Published:** 2021-10-22

**Authors:** Ludvig Löwemark, Alice Chien-Yi Liao, Yu-Hsuan Liou, Shital Godad, Ting-Yi Chang, Alexander Kunz

**Affiliations:** Department of Geosciences, National Taiwan University, No 1. Sec. 4 Roosevelt Road, P.O. Box 13-318, Taipei, 106 Taiwan

**Keywords:** Environmental chemistry, Environmental monitoring, Geochemistry, Pollution remediation, Biogeochemistry, Environmental sciences

## Abstract

Detecting clandestine, intermittent release of heavy metal pollution into natural and man-made water ways is challenging. Conventional chemical methods are both labor intensive and expensive. A recent approach combining ion-exchange resins with the capabilities of X-ray fluorescence core scanners (XRF-CS) therefore is of great interest. In short, ion-exchange resin is deployed in the water using small sachets, the resin is then collected, dried, filled into sample holders and scanned using XRF-CS. Ion-exchange resins take up heavy metals in proportion to the concentration in the ambient water, with a correlation coefficient (R^2^) between concentration and XRF-CS counts better than 0.96 for most elements. However, a number of parameters influence the measurements. Different drying methods introduce differences in the XRF counts because of lattice bound water, resin shrinkage, and disaggregation of the resin particles. Furthermore, the newly developed sample carrier, which was constructed using 3D printed polymers, contains trace amounts of elements that may influence the sample measurements through edge effects and secondary fluorescence. In the tested sample carrier materials, substantial levels of Cr, Fe, Co, and Zn were detected, while Ca, Ti, Ni, Cu, Ga showed variable levels. Ba, Tl and Bi show very low levels, and Pb is only of importance in the PLA carrier. It is therefore necessary to streamline the analysis-process to ensure that the variations in sample treatment and drying and filling methods are minimized. It is also recommended that only spectra from the center of the compartments are used for the evaluation to avoid edge effects caused by secondary fluorescence of metals in the compartment walls. Although the technique of using ion-exchange resin sachets and XRF-CS analysis is only semi-quantitative, it is a cost effective and fast way to monitor large areas for environmental pollution, and the new sample carrier greatly contributes to make the process faster and less error prone.

## Introduction

In the 1990’s, the growing number of long, marine and lacustrine sedimentary records retrieved for paleoclimatic research highlighted the need for fast and non-destructive analytical methods. As a result, a number of core logging and scanning techniques were developed^1^. In particular, several different X-ray fluorescence scanners were independently developed that allowed downcore chemical variability in fresh, untreated, sediment cores to be rapidly assessed at sub-millimeter resolution see overview by^2^. Although the technical solutions differ between the three main players (Avaatech, Geotek, and Itrax), the basic principle remains the same: a split sediment core is shifted relative to an X-ray source, and a detector reads the X-ray fluorescence emitted from the sediment surface. This technique is inherently semi-quantitative at best, but it allows for an extremely fast assessment of downcore variations in elements typically ranging from Mg/Al to U; analyses that would take weeks to months using conventional geochemical methods can be obtained within hours. Obvious limitations come from the fact that the measurements are performed directly on the moist sediment and consequently no universal standards can be employed. However, the semi-quantitative nature of the results can often be overcome by utilizing ratios of various elements instead of absolute concentrations cf.^3,4^. Despite being explicitly developed for unlithified, moist sediment cores, it didn’t take too long until the community started experimenting with applying the XRF core scanning technique to hard rock samples such as sandstones^5^ or speleothems^6^, tree rings^7^, frozen samples^8^, or powdered samples^9^. Analyzing powdered samples on an XRF core scanner requires some sample preparation and the loading of samples onto a specially designed sample carrier e.g.,^10^. This to some extent counteracts the XRF core scanners advantages, i.e. being fast and non-destructive.

In many cases, however, the ability to process large quantities of powdered or granular samples in a short time with a minimum of sample preparation outweighs the disadvantage of only obtaining semi-quantitative data. A recent example is the application of ion exchange resins to assess environmental pollution in rivers and drainage systems^11^. Because the resin takes up ions from the water in proportion to the concentration in the water, the concentration of the elements in the resin can be used as a direct measure of the pollution in the water. Huang et al.^11^ applied this to a partly industrialized former farmland area in central Taiwan to pinpoint hotspots of heavy metal pollution related to the factories in the area. In short, the resin was placed in sachets that were deployed across the drainage system for a number of days. The sachets were then collected, rinsed and dried, and the resin was placed in purpose-built cuvettes that could be lined up and scanned with the XRF core scanner. This allowed a large area to be assessed, and contamination hotspots related to industries scattered around the farmland could be visualized using geographical information systems. A similar approach was followed by Pan et al.^12^, but using a portable XRF.

The bottleneck of using the resin sachet approach to monitor larger areas over time is the handling of the cuvettes. Even though aids can be developed to help reduce the time needed to fill the cuvettes and reduce the risk of cross-contamination cf.^13^, the loading of the resin into large numbers of small, individual cuvettes, and loading these onto the rail of the XRF core scanner is time consuming, error prone, and the small size of the cuvettes make them easy to tip over or get mixed up.

The aim of this study is to describe and evaluate a novel sample carrier that streamlines the filling of the resin, limits the risk of cross-contamination, sample mix-ups and sample loss, and significantly reduces the time needed to fill and rinse the sample carrier. We also evaluate the potential effects of different sample carrier materials, the influence of matrix effects when scanning ion exchange resins that largely consist of XRF transparent elements, and how different methods of drying the resin after deployment affect the signal.

## Material and methods

### Sample carrier

**Figure 1:**
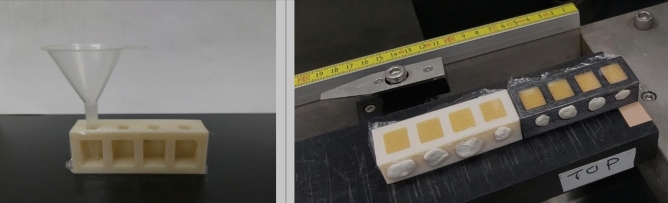
Sample carrier for granular or powdered samples. (**A**) The compartment openings are covered with XRF film and then the carrier is flipped 90 degrees, filled with resin, sealed, flipped back, and then mounted onto the rails of the Itrax XRF Core Scanner. (**B**) The whole carrier is scanned in one procedure, but only spectra from the middle of each compartment are used to avoid edge effects and bias from the carrier material.

The sample carrier was designed using CAD and produced using 3D printing techniques (Additive Intelligence Co., Ltd., Taipei). Several prototypes were produced using generic polymers to test the functionality of the carrier. Finally, three smaller sample carriers using different polymers were produced to assess the influence of different sample carrier materials on the XRF analysis. The polymers used were polylactic acid (PLA), acrylonitrile styrene acrylate (ASA), and polyethylene terephthalate glycol (PET-G). The final full length sample carrier (PLA) consists of a 26 cm long bar with rectangular compartments (width 15 mm, length 10 mm, depth 10 mm) for the resin on the top and filling holes on the side (Fig. 1). During filling, the rectangular openings were covered with an XRF film (e.g. Mylar or polypropylene) and the carrier was flipped 90 degrees to allow a funnel to be attached to the filling hole. After the compartments were filled, the holes were sealed using mounting putty and the carrier was flipped back in its original position. The carrier was then placed onto the XRF core scanner rail and all compartments were scanned in one procedure. The XRF core scanner used was the machine in the NTU Itrax XRF Core Scanner Facility at National Taiwan University, equipped with a custom-built CUBE-ASIC XRF detector (energy resolution 130.2 eV FWHM, count rate 20.000 s^−1^) and an analytical foot print of 4*0.2 mm. The carrier was scanned using a Mo tube with a step size of 0.2 mm, an exposure time of 5 s, and a voltage and current of 30 kV and 50 mA, respectively. The obtained spectra were post-processed using the built-in Qspec program, and spectra related to the compartment walls and close to the edges of the compartments were separated; only spectra from the central part of the resin-filled compartments were used for analysis of the resin.

### Sample carrier composition

To assess the potential influence of elements present in the sample carrier polymers, the counts recorded from the carrier walls were compared to the counts measured over unused ion-exchange resin (blanks). Because the whole sample carrier is scanned continuously in one scan-procedure, the spectra recorded from the compartment walls can be used to assess the presence of metals in the different polymers used in the 3D printing of the carrier. The exact chemical composition of the polymers used goes far beyond the scope of this study, but by comparing the XRF spectra obtained from the sample carrier’s walls to the spectra of the unused ion-exchange resin (blanks), a reasonable assessment of the various elements present in the different polymers can be made. Twenty spectra from the middle of the walls separating the sample carrier’s compartments, and fifty spectra from the blank were used to assess the average element ratio between sample carrier and resin. Elements showing elevated values relative to the background of the resin could potentially bias spectra near the edges of the compartments.

### Ion-exchange resin

For this study, the cation-exchange resin Amberlite TM IR-120 (Rohm and Hass, USA) cf.^14–16^ was chosen because it has already been deployed in at least two studies using resin sachets and XRF analysis^11,12^. About 4 g of the resin was filled into polyethylene (PE) net sachets that were then placed in standardized solutions prepared from Merck Certipur® ICP standards (Product numbers 1.11355.0100 and 1.70363.0100). The solutions used contained 10 ppm or 50 ppm of the following elements: Li, B, Na, Mg, Al, K, Ca, Ti, Cr, Mn, Fe, Co, Ni, Cu, Zn, Ga, Sr, Ag, Cd, In, Ba, Tl, Pb, Bi. However, Li, B, Na, Mg, and to some extent Al, cannot be detected by the Itrax XRF Core Scanner, and S and K are present in the ion-exchange resin itself. Moreover, the elements Ag, Cd, and In showed poor reproducibility (as determined through duplicate scans) at the relatively low concentrations used in this experiment Consequently, only the following fifteen elements are discussed: Ca, Ti, Cr, Mn, Fe, Co, Ni, Cu, Zn, Ga, Sr, Ba, Tl, Pb, Bi.

After deployment, the sachets were rinsed with deionized water and dried. Three different methods of drying were tested: oven drying at 50 °C, freeze drying, and drying at room temperature under a fan. For the evaluation of the influence of different drying methods, three duplicate samples subjected to a 10 ppm test solution were used. The drying tests were then scanned on the XRF core scanner using the old cuvettes cf.^11^. To assess changes to the resins caused be the three drying methods used, 400–500 resin balls were scrutinized under microscope and the relative shrinking of the resin was determined using the program Image J cf.^17^. Kruskal–Wallis test was used to test if there are statistically significant differences in the results between drying methods for each element. Additionally, Scheffe’s test was performed to identify which drying methods yield significantly different results and which ones yield similar results. For both tests the null hypothesis was that the drying methods produce different results. As a significance threshold we used a two-tailed *P*-value < 0.05. All statistical analyses were done using R version 4.1.0^18^ and DescTools R package version 0.99.42^19^.

In order to estimate the optimal duration of resin deployment, a 50 ppm solution was used, and resin sachets were exposed for increasingly longer durations (2–12 days) to assess when saturation was reached. The sachets were then scanned and the optimal time determined in order to ensure that adsorbed levels neither continued to increase nor decrease, as the time in the solution increased. The main purpose of this test was to establish a reasonable time for the deployment of resin sachets in natural waters.

### Resin calibration test

The adsorption of ions to the ion-exchange resin from a solution, and the calibration of the XRF counts of the resin relative to the concentrations of elements in the solution has been thoroughly evaluated in earlier studies^11,12^. However, these calibrations were developed for solutions containing primarily divalent cations. In natural waters the presence of monovalent matrix ions can influence the uptake of divalent cations. It is therefore of importance to develop calibration solutions that correspond as closely as possible to the water studied. In this study, only a basic verification of the calibration was performed. In short, to test the calibration, resin sachets were immersed in (0.05, 0.1, 0.2, 0.5, 1, 2, and 5 ppm) solutions for two hours, dried and scanned using the Itrax XRF core scanner. The sample solution was analyzed and the uptake calculated as the difference between original concentration and solution concentration after the experiment (See supplementary material).

## Results

### Sample carrier filling, scanning and cleaning

A comparison between the novel sample carrier and the cuvettes previously used for analyzing powdered and granular samples revealed several benefits. The new sample carrier reduces the time needed for filling the resin into the compartments to less than half, compared to the conventionally used individual cuvettes. It also significantly reduces the risk of mixing up samples as the compartments are fixed on the carrier, and, the wide spacing of the filling-holes in combination with the use of a funnel for inserting the resin into the compartments on the carrier reduces the risk of cross-contamination. Emptying and cleaning the carrier is easier, and the resin can easily be recovered for other analyses if needed. Because the resin is confined inside the carrier, the risk of accidentally losing all or some of the samples during transport in the lab is also considerably reduced.

### Sample holder material and edge effects

**Figure 2:**
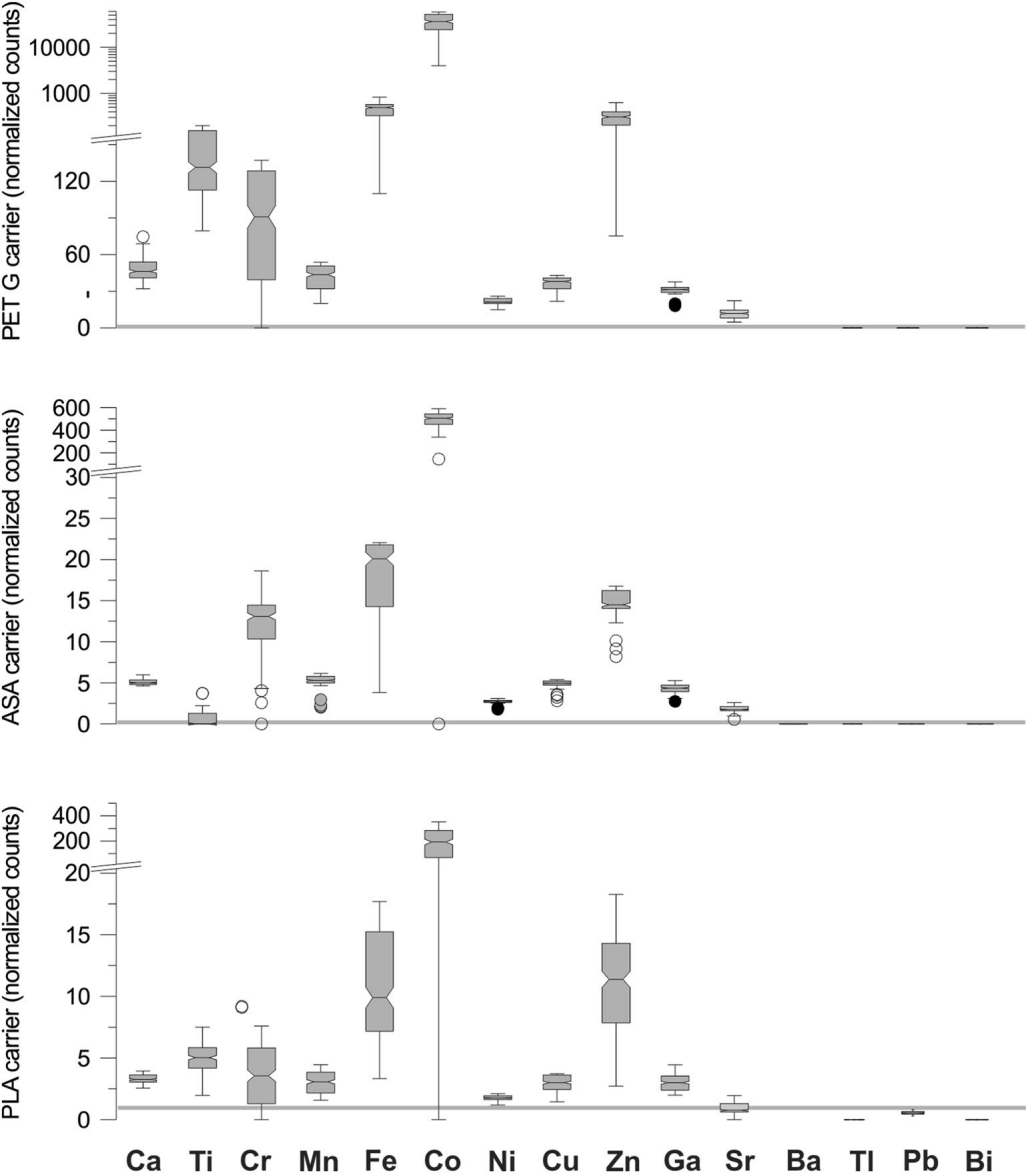
Comparison of sample-carrier-to-resin-blank element ratios for the different polymers used in this study. High ratios indicate that detectable levels of the element is present in the sample carrier materials. Noteworthy levels of Cr, Fe, Co, and Zn were observed in all three polymers used. Dotted gray line marks the 1:1 ratio, indicating no difference between sample carrier and resin blank.

**Figure 3:**
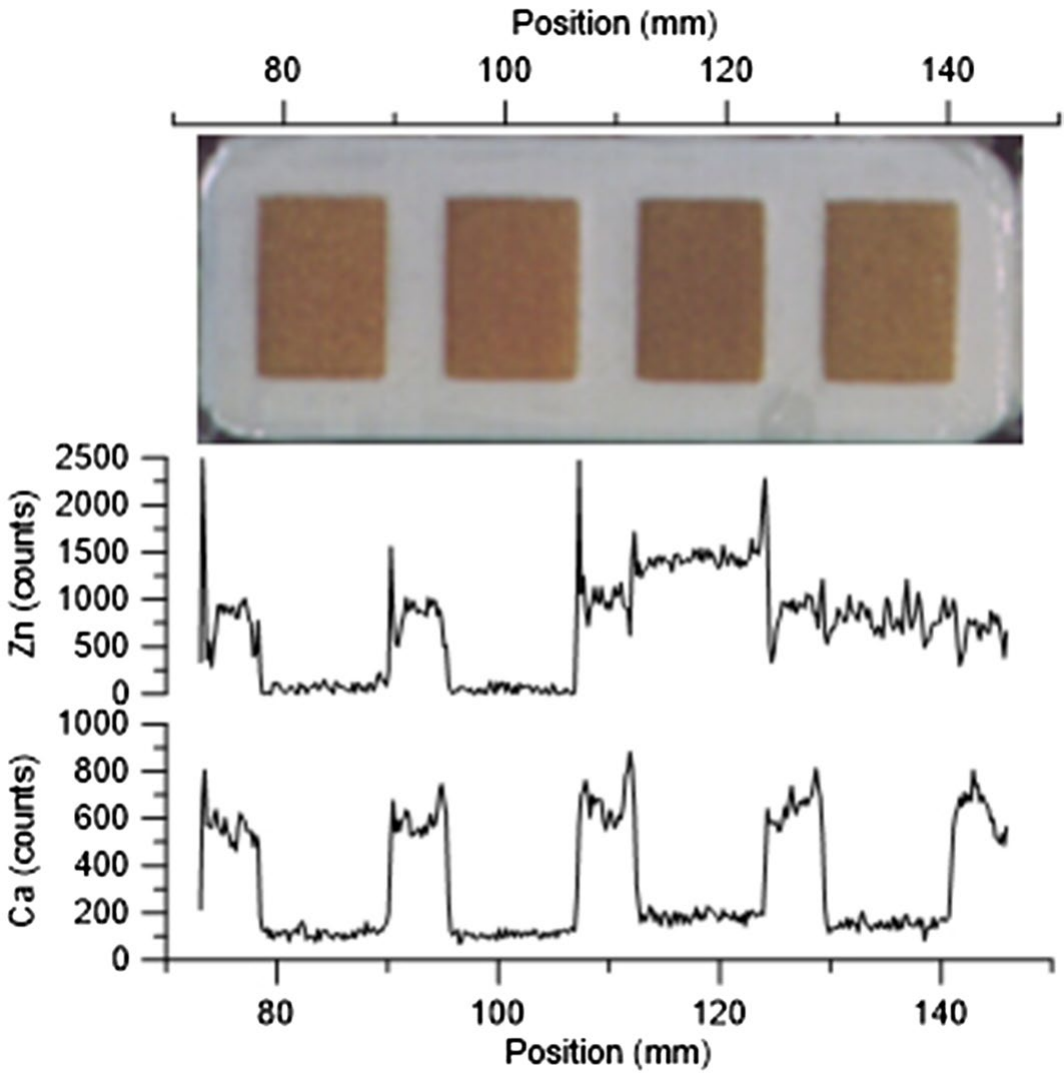
Optical image and XRF scan results for Ca and Zn in one of the three sample carrier prototypes (ASA). The Ca counts show strongly elevated levels over the compartment walls, compared to the ion-exchange resin. The Zn counts show, as expected, higher values in the resins that were immersed in the test solution, but also distinct peaks at the boundaries of the the sample carrier compartment walls. The carrier was loaded with (left to right) untreated resin, resin exposed to deionized water, resin exposed to 10 ppm solution, resin exposed to < 10 ppm solution.

The XRF scans of the three tested prototypes revealed two important features. First, the XRF counts detected over the compartment walls showed strongly elevated levels for several elements, indicating that the polymers used in the sample carrier contained substantial levels of these elements. High to extremely high levels of Cr, Fe, Co, and Zn were detected, while Ca, Ti, Ni, Cu, Ga showed intermediate to high but variable levels relative to the unused ion-exchange resin. Ba, Tl and Bi show very low levels, well below the levels of the unused ion-exchange resin. Pb displayed low levels in the ASA and PET-G carriers, and is only of importance in the PLA carrier (Fig. 2). The resin itself contains high levels of S and K, rendering the assessment of the presence of these elements in the polymer useless (Supplementary data). Second, for some elements, substantially higher counts were observed from the analysis of the ion-exchange resin directly next to the compartment walls. For example, over the compartment walls Ca levels are high, even considerably higher than in the resin exposed to the 10 ppm solution, but there are also peaks in the ion-exchange resin directly next to the walls. Similarly, Zn shows low levels in the blanks, high levels in the resin exposed to the 10 ppm solution and in the compartment walls, but also sharp peaks in the resin just next to the walls (Fig. 3). These peaks likely are caused by secondary X-ray fluorescence emitted from elements in the walls of the sample carrier.

### Influence of drying method

**Figure 4:**
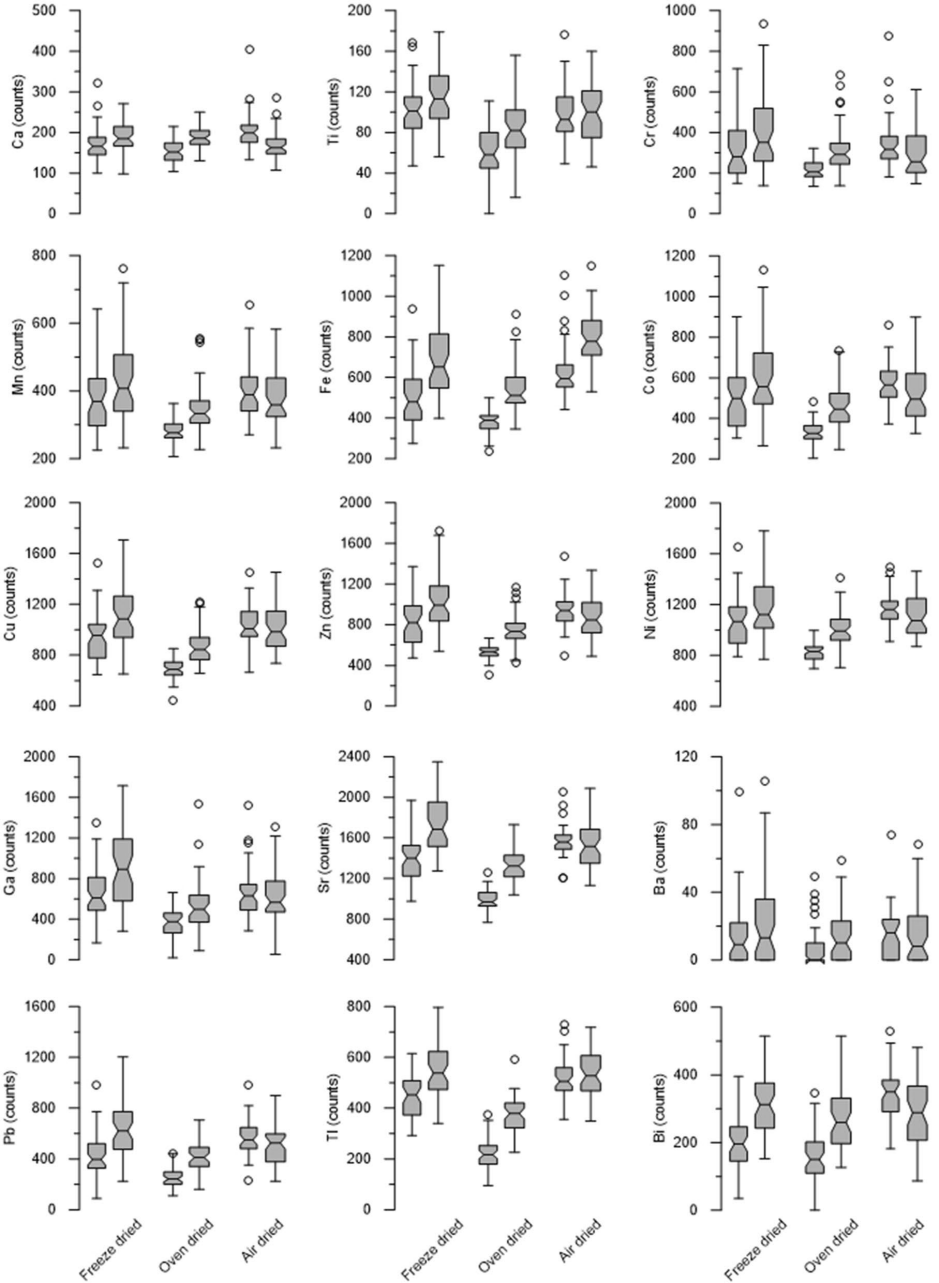
Box plots showing the measured element counts of the three drying methods for the fifteen relevant elements in the test solution. Generally, freeze dried samples show higher counts, while the oven dried samples display a smaller variance in most element counts.

Both signal strength and variance show substantial differences between the samples dried using the three different methods (freeze drying, oven drying, air drying) (Fig. 4). In general, freeze drying and air drying show higher signal strength (more counts) but also larger variance. Oven dried resin samples showed considerably lower signal strength (fewer counts) but also lower variance resulting in a more focused signal. Evaluations were based on 30 spectra from the middle of the sample, thus excluding 10 spectra on each side to avoid edge effect and possible bias by the sample carrier. The results of the Kruskal–Wallis test showed *P*-values < 0.05 for all drying methods in all elements, meaning that there are statistically significant differences between the drying methods. The results of the post-hoc Scheffe’s test don’t show a clear pattern, but a trend can be observed indicating that air drying and freeze drying lead to similar results, whereas oven drying leads to differences compared to air and freeze drying. Quantitative evaluation of the size of the resin balls under microscope revealed that freeze-dried resin-balls were on average about 10% smaller than air-dried resin, while oven-dried resin balls were about 2% smaller than air dried. This suggests that freeze drying removes more of the moisture from the resin, leading to stronger shrinkage.

### Resin element uptake parameters

**Figure 5:**
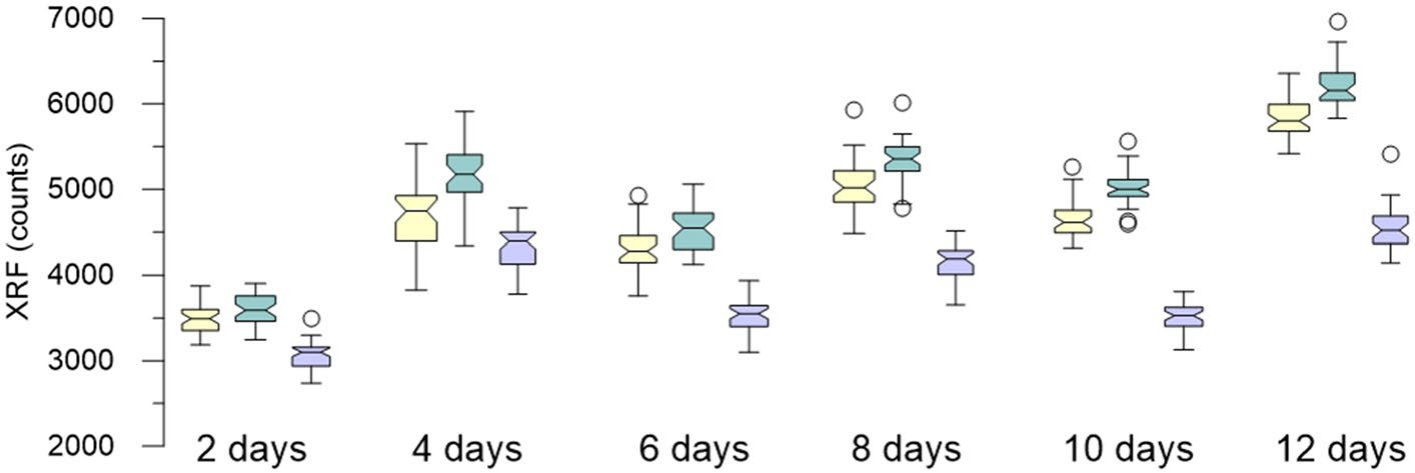
Examples of the effect of different adsorption times in a 50 ppm solution over two to twelve days. Yellow = Ni, Turquois = Zn, Powder Blue = Pb.

The resin sachets were analyzed after being exposed to a 50 ppm test solution for two to twelve days, in two-day increments. After the fourth day, most elements reached a plateau where little increase was seen as the adsorption times were increased (Fig. 5). However, for some elements, such as Ni or Zn, the adsorption to the resin continued until the twelfth day.

### Calibration test

**Figure 6:**
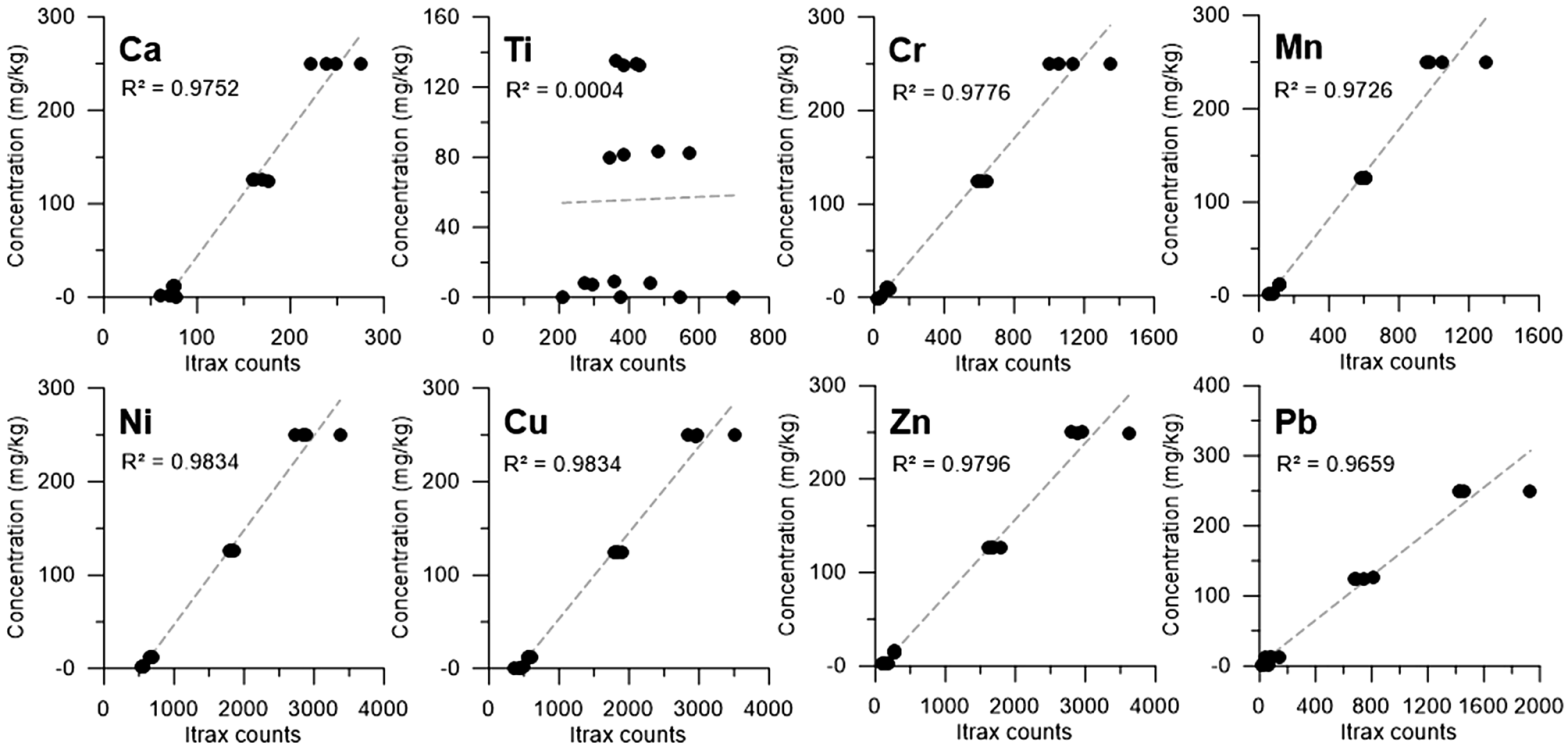
Cross plots for eight selected elements used to test the calibration of Itrax-CS counts to concentrations. Except for Ti, good correlations (R^2^) above 0.96 were achieved.

The calibration experiment was performed to evaluate the amount of elements adsorbed onto the resin. Eight elements related industrial activities (Ca, Ti, Cr, Mn, Ni, Cu, Zn, Pb) were selected. To procure concentrations we followed the approach in Huang et al.^11^ and applied the mass balance Eq. 1 (Supplementary data). Except for Ti, the concentrations calculated using Eq. 1 and XRF-CS counts indicate good linear correlation with R^2^ > 0.96 (Fig. 6).

## Discussion

### Evaluation of the efficiency of the new sample carrier design

Although ion-exchange resin can be efficiently studied using a handheld XRF^12^ or the cuvettes designed for peat and soil samples^11,13^, when sample volumes increase, the need for a streamlined processes becomes apparent. The new sample carrier has several advantages. First, the time needed to load and clean the carrier is considerably reduced compared to cuvettes or other open sample holders. Second, the filling of the carrier from the side-holes using a funnel largely eliminates the risk of cross-contamination and diminishes the risk of sample mix-ups to a minimum. In addition, because the resin is locked-in by the XRF film and the seals of the filling holes, the resin can be used for other analysis and the risk of losing samples because the sample holder tips over or slips during transport is greatly reduced. Consequently, the new sample carrier allows for an increased through-put with less-risk of sample mix-ups or sample loss. Operating the new sample carrier does not require any special skills and the operating protocol can be taught in minutes, lending it particularly suitable for citizen-science projects involving students or other members of the general public.

### Sample holder geometry and material issues

The analysis depth, the depth from which no primary or secondary fluorescence can escape, varies from just a few micrometers to several cm depending on material and X-ray source used^20^. Because the ion-exchange resin is largely transparent to the X-ray spectrum used to illuminate the samples, the analytical depth is not reached. This means that the XRF spectra measured over the resin may be influenced by fluorescence emitted from the sample carrier, especially near the compartment walls. Close to the compartment walls, primary fluorescence from elements in the resin may reach the detector, and fluorescence from the elements in the wall may irradiate elements in the ion-exchange resin to cause secondary fluorescence, resulting in biased values. The spectra measured directly from the compartment walls showed that all the tested polymers contain substantial amounts of certain elements relative to the unused resin (blanks). To avoid the potential influence of these elements in the sample carrier, it is recommended to discard about ten spectra (2 mm) on each side of the sample. For example, a closer look at the distribution of counts for Zn reveals elevated counts near the edge of the compartment, although these elements are not detected in the sample carrier itself (Fig. 3). These elevated counts are best explained by secondary fluorescence in the resin caused by primary fluorescence from elements in the sample-carrier walls cf.^20–23^, resulting in additional excitation of elements such as Ni, Cu, and Zn. This secondary fluorescence induced by the irradiation from X-rays emitted by the walls contribute to the total count-rate and can result in biased values for certain elements. The effect is largest directly next to the compartment walls and can be avoided by ignoring about ten spectra closest to the walls (about 2 mm of the sample). For analysis of ion-exchange samples or other granular materials largely consisting of organic materials, such as peat, it is therefore recommended that only 20–30 spectra from the center of the sample are used.

**Figure 7:**
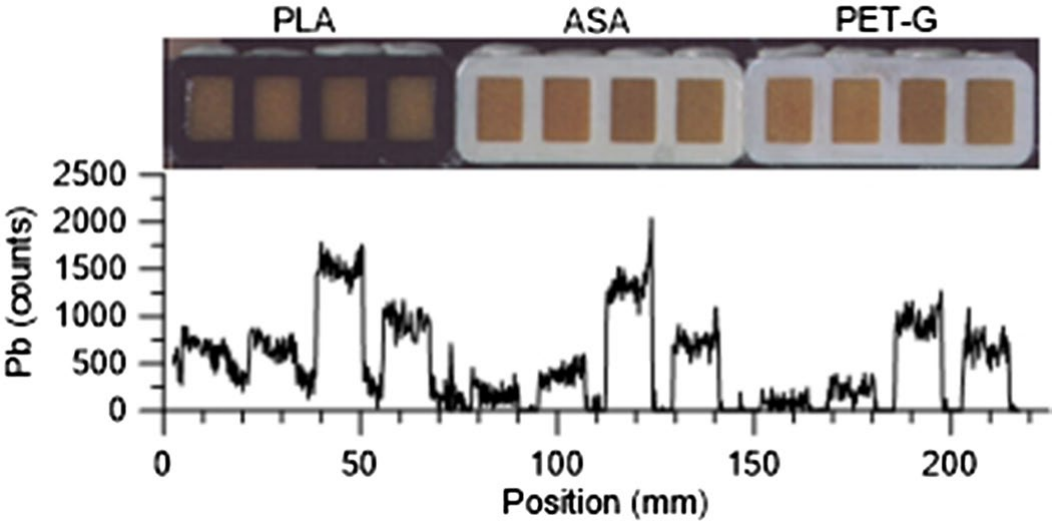
Comparison of the Pb signal between the three prototypes tested (Black = PLA, middle white = ASA, right white = PET-G). Note the considerably higher levels measured over the compartment walls for the PLA carrier compared to the levels in the ASA and the PET-G carriers. All carriers loaded with (left to right) untreated resin, resin exposed to deionized water, resin exposed to 10 ppm solution, resin exposed to < 10 ppm solution.

However, in some cases there is an obvious and strong influence by the sample carrier that is not readily identified by looking at the ratio between sample-carrier walls and resin. For example, comparing the counts for Pb from the three prototypes tested using four differently pretreated ion-exchange resins (untreated resin, resin exposed to deionized water, resin exposed to 10 ppm reference solution, and resin exposed to a diluted reference solution (< 10 ppm)) clearly shows that counts are generally higher in the PLA-carrier (black) than in the two other carriers (Fig. 7). In the PLA carrier, Pb levels in the reference resins (untreated and deionized water) show high count levels reaching almost half of the values measured over the compartment walls. In contrast, the ASA and PET-G carriers show considerably lower counts, with almost zero counts over the carrier walls. Moreover, the Pb levels are also higher in the PLA carrier compared to the ASA and PET-G carriers for the compartments containing resins treated with the test solution. As the resin comes from the same sachets, the differences in Pb counts between the carriers must be related to the composition of the carrier 3D printing material. Because the ion-exchange resin primarily consists of carbon compounds (polystyrene), the mass attenuation coefficient is very low cf.^24^, and the resin is therefore largely transparent to the X-ray spectrum emitted from the Mo-tube. Consequently, the analysis depth is not reached, and for some elements, even XRF emitted from the base of the sample-carrier compartment can influence the signal registered by the detector. Relative excitation efficiency of different elements strongly depends on the intensity and spectrum of the X-ray source, but the relationship is not linear and varies from element to element^25^. This means that some elements, such as Pb, may fluoresce strongly from the base of the sample carrier even though covered by ion-exchange resin. Consequently, in a sample carrier that contains high levels of heavy elements in the polymer, a general bias towards higher counts may be observed, and needs to be accounted for when identifying possible pollution sources.

In the case of the studied three sample carriers, we prefer the PLA-carrier, as the polymer to ion-exchange resin ratio is generally lower for most elements. However, care must be exercised when interpreting Pb as even low levels in the sample carrier polymer can influence the outcome at low concentrations in the ion-exchange resin.

### Drying

The observed discrepancies in XRF counts between the different drying methods are likely due to differences in two parameters related to the drying process itself: the content of lattice bound crystalline water in the ion-exchange resin, and density differences caused by shrinking of the resin during the drying. Basically, a drying method that removes more crystalline water from the resin matrix leads to less attenuation of emitted XRF from the elements in the resin. The influence of interstitial water on the XRF intensities have been well-documented for moist sediment cores e.g.,^25,26^. In dried resin samples the absolute differences in water content should be small. However, the large analytical depth of the ion-exchange resin means that the XRF signal detected depends on fluorescence emitted from a much thicker layer of the resin sample compared to sediment samples. This amplifies differences because of the attenuating effect of water on XRF radiation emitted deeper in the resin. Since the effect of XRF adsorption by interstitial and lattice-bound water affects each element differently, not all elements would be attenuated equally.

Moreover, resin studies have shown that the volume of the resin may shrink when the resin is dried, and also that there are changes in the adsorption of certain elements to the resin due to the drying^27,28^. As a consequence, the concentration of adsorbed elements would increase in a resin that is dried in a way that cause more shrinking relative to other drying methods, and a denser resin would yield higher counts. For most of the elements, highest counts were obtained from the freeze-dried resin, and the lowest counts from the oven-dried resins. This suggests that freeze-drying is the most efficient drying method, removing more water than the other methods and possibly also leading to more shrinkage of the resin. This is also confirmed by the microscopic analysis that showed an about 10% smaller resin ball-size for freeze-dried resin compared to the air-dried resin. Smaller resin balls means higher concentrations of the elements taken up by the ion-exchange resin.

A further factor related to the drying process is the influence of the XRF film (Mylar) used to cover the sample carrier. In sediment cores, several studies have noted the formation of a thin water film underneath the XRF film. This film can have a substantial effect attenuating the XRF signal of especially lighter elements^4,25,29^. The influence of the XRF film on the scanning of resin samples is likely minor, as little mobile water should be present.

However, there were also substantial differences in the variance of the counts between the different drying methods. The XRF counts from the oven-dried resin show considerably smaller variance than both freeze dried and air dried resins. For the freeze dried resins, the freezing may cause the resin particles to crack, thereby changing both their stacking pattern and their surface texture. It has been demonstrated that sample surface can have a substantial influence on the XRF signal^25^. It is possible that the different drying procedures could lead to differences in the aggregation of resin grains, thus leading to variations in the smoothness of the sample surface, thereby inducing the observed differences in the variance. It has been demonstrated that ion-exchange resins that are exposed to wet-dry cycles may induce desorption of certain elements due to the shrinking of the resin^30^. We speculate that the three different drying methods applied in this study may have resulted in small differences in adsorption–desorption of different elements, giving rise to the variations in XRF counts observed.

In summary, care need to be taken to streamline the drying protocol to ensure that all samples are dried in the same way and that differences in water content are minimized. In this way, results from different water sources, or time series of pollution, will be comparable.

### Uptake of ions into resin

The application of resin bags or sachets to address the presence and distribution of various elements in the natural environment is not new e.g.,^31–33^, and the adsorption times needed under laboratory conditions have been thoroughly tested^12,34^. The tests performed in this study aimed at determining a reasonable time frame that would allow the resin sachets to be deployed in natural waters without putting too much pressure on the exact timing on the retrieval of the sachets. As most elements reached a plateau in the adsorption to the resin after about four days and no desorption was observed even after 12 days, a reasonable compromise is to leave the sachets in the water for a week, as this allows for the monitoring of larger areas without exhausting the resources deploying and collecting the resin sachets.

### Calibration of XRF counts

The calibration of XRF counts measured from the ion-exchange resin used, to solution concentrations have been extensively tested in previous studies^11,12^. Here only a minor test to confirm previous results was performed. The output of this method demonstrated good correlation between XRF-counts and absolute concentrations for the tested elements, except for Ti. The poor calibration for Ti likely is related to the fact that Ti would not be expected to be present as cationic species under normal conditions, thereby possibly influencing the uptake by the ion-exchange resin. Although Ti is generally considered a conservative species and is not abundant in dissolved form in natural waters^35–37^, the element is often used to normalize other environmentally relevant elements^38,39^. Caution must therefore be exercised when interpreting ion exchange resin based results of changes in Ti levels in natural waters.

## Conclusions

The combination of ion-exchange resin and XRF core scanners is a cost effective method to assess heavy metal pollution in natural waters and to geographically pinpoint pollution sources. The method is inherently semi-quantitative, but the fast turn-over and the low cost makes it an attractive alternative for environmental monitoring. Based on the XRF core scanner analysis of a novel sample carrier for ion-exchange resins deployed in aquatic solutions using small sachets, a number of conclusions can be made.

The new sample carrier can be filled and cleaned faster, and also reduces the risk of cross-contamination and sample loss. However, the sample carrier material can influence the measurements for certain elements, and close to the compartment walls, edge effects may result in abnormal counts. It was also demonstrated that different methods of drying the ion-exchange resin may result in shifts in both count levels and variance. The optimal deployment times of ion-exchange resin sachets to monitor natural waters is about a week.

In summary, the use of ion-exchange resin sachets and XRF Core scanners is a fast and cost efficient way to monitor pollution in natural and constructed water ways, but care must be taken to ensure that the potential influence of elements in the sample carrier is accounted for. Moreover, care must be taken to ensure that all measurements are performed under identical conditions in terms of drying methods, sample volume and XRF core scanner settings.

## Supplementary Information

Supplementary Information.
