# Supplementary material for: Potential and pitfalls of XRF-CS analysis of ion-exchange resins in environmental studies
Source: Sci Rep. 2021 Oct 22;11:20941. doi: 10.1038/s41598-021-00446-9 (PMC8536750; doi:10.1038/s41598-021-00446-9)
Supplement: Supplementary file 1 — Supplementary Information. [file 41598_2021_446_MOESM1_ESM.docx]

Supplementary Information

Resin – Calibration Test

To carry out the calibration test we used four standard solutions by diluting commercial stock solution (Merck ICP multi-element standards) of Mn, Cr, Ni, Cu, Zn, Pb, Ca, Ti (Ca and Ti were added as these elements are found regularly into the natural water) with deionized water according to the Environmental Protection Act (EPA) effluent standard guidelines of Taiwan. To further make it cost effective, the standards were prepared using beaker instead of a Jar-tester. Magnetic stirrers were used to simulate different flow rates as in the natural environment. The standard solutions were prepared using four stirring speeds (stirring speeds 50 rpm, 100 rpm, 150 rpm and 200 rpm). These standard solutions were separated into 16 batches of 1L volume. The resin sachets were immersed into each batch for 2 hours at room temperature. For analysis of metal concentration through ICP-OES, 5 ml of each solutions were used. To obtain the concentration of the resin reference standard, following equation (1) was used which follows law of mass balance from initial and final concentration of the standard solutions.

Cm = ((C_0_ – C_t_)*L/ W )*1000 ---- (1)

Cm = Concentration of resin reference standards (mg/kg)

C_0_ = initial concentration of metal in the standard solutions (mg/L)

C_t_ = final concentration of metal in the standard solutions (mg/L)

L and w = volume (L) of the standard solutions and weight of resin (g).
